# Managing Remote Caregiving: Orchestrating a Care Network for People With Dementia via Digital Communication

**DOI:** 10.1093/geroni/igaf122.717

**Published:** 2025-12-31

**Authors:** Patricia Heyn

**Affiliations:** Marymount University, Arlington, Virginia, United States

## Abstract

The growing reliance on digital communication tools has transformed caregiving, particularly for those managing care from a distance. This autoethnographic reflection examines the complexities of orchestrating a remote care network for a loved one, utilizing WhatsApp and other digital platforms to coordinate with healthcare providers in real time. As more adults navigate the sandwich generation—balancing professional responsibilities while caring for both children and aging parents—many assume the dual role of care manager and emotional anchor, ensuring their loved one’s medical needs, well-being, and support systems remain integrated despite physical distance. This narrative explores the benefits of digital communication in caregiving, including immediate access to medical updates, medication management, and emergency decision-making, alongside the emotional and logistical burdens of long-distance caregiving. Key themes include the role of technology in coordinating multidisciplinary care teams, the challenges of remote medical advocacy, and strategies for balancing work and caregiving responsibilities. The discussion also addresses the limitations of digital caregiving, highlighting gaps in telemedicine support and the burden on long-distance caregivers. By integrating personal insights, digital health data, and multimedia documentation—including photos, videos, and shared decision-making moments—this abstract contributes to the broader discourse on technology-driven caregiving, caregiver stress, and the evolving role of digital health tools. As gerontologists, we have long studied the aging experience, yet today, we find ourselves living it firsthand—navigating the very challenges we research and advocate for, as our personal and professional worlds converge in the realities of caregiving.

